# Relationship Between Hg Speciation and Hg Methylation/Demethylation Processes in the Sulfate-Reducing Bacterium *Pseudodesulfovibrio hydrargyri*: Evidences From HERFD-XANES and Nano-XRF

**DOI:** 10.3389/fmicb.2020.584715

**Published:** 2020-10-14

**Authors:** Marie-Pierre Isaure, Marine Albertelli, Isabelle Kieffer, Rémi Tucoulou, Melina Petrel, Etienne Gontier, Emmanuel Tessier, Mathilde Monperrus, Marisol Goñi-Urriza

**Affiliations:** ^1^Université de Pau et des Pays de l’Adour, E2S UPPA, CNRS, MIRA, IPREM, Pau, France; ^2^FAME-UHD, BM16 Beamline, European Synchrotron Radiation Facility (ESRF), BP220, Grenoble, France; ^3^CNRS, IRD, Irstea, Météo France, OSUG, FAME, Université Grenoble Alpes, Grenoble, France; ^4^ID16B Beamline, European Synchrotron Radiation Facility (ESRF), BP220, Grenoble, France; ^5^Bordeaux Imaging Center UMS 3420 CNRS – US4 INSERM, Université de Bordeaux, Pôle d’imagerie Électronique, Bordeaux, France; ^6^Université de Pau et des Pays de l’Adour, E2S UPPA, CNRS, MIRA, IPREM, Anglet, France

**Keywords:** Hg, microorganisms, methylmercury, methylation, demethylation, nano-XRF, HERFD- XANES

## Abstract

Microorganisms are key players in the transformation of mercury into neurotoxic methylmercury (MeHg). Nevertheless, this mechanism and the opposite MeHg demethylation remain poorly understood. Here, we explored the impact of inorganic mercury (IHg) and MeHg concentrations from 0.05 to 50 μM on the production and degradation of MeHg in two sulfate-reducing bacteria, *Pseudodesulfovibrio hydrargyri* BerOc1 able to methylate and demethylate mercury and *Desulfovibrio desulfuricans* G200 only able to demethylate MeHg. MeHg produced by BerOc1 increased with increasing IHg concentration with a maximum attained for 5 μM, and suggested a saturation of the process. MeHg was mainly found in the supernatant suggesting its export from the cell. Hg L_3_-edge High- Energy-Resolution-Fluorescence-Detected-X-ray-Absorption-Near-Edge-Structure spectroscopy (HERFD-XANES) identified MeHg produced by BerOc1 as MeHg-cysteine_2_ form. A dominant tetracoordinated βHgS form was detected for BerOc1 exposed to the lowest IHg concentrations where methylation was detected. In contrast, at the highest exposure (50 μM) where Hg methylation was abolished, Hg species drastically changed suggesting a role of Hg speciation in the production of MeHg. The tetracoordinated βHgS was likely present as nano-particles as suggested by transmission electron microscopy combined to X-ray energy dispersive spectroscopy (TEM-X-EDS) and nano-X ray fluorescence (nano-XRF). When exposed to MeHg, the production of IHg, on the contrary, increased with the increase of MeHg exposure until 50 μM for both BerOc1 and G200 strains, suggesting that demethylation did not require intact biological activity. The formed IHg species were identified as various tetracoordinated Hg-S forms. These results highlight the important role of thiol ligands and Hg coordination in Hg methylation and demethylation processes.

## Introduction

Methylmercury (MeHg) is a serious threat as it is a neurotoxin bioaccumulated and bioamplified in food webs. Mercury methylation is a biotic process mainly driven by anaerobic microorganisms including sulfate-reducing bacteria, iron reducing bacteria, and methanogens (Gilmour et al., 2013; Gionfriddo et al., 2016). Recently, other microorganisms were also shown to be able to methylate Hg (Jones et al., 2019; Azaroff et al., 2020; Villar et al., 2020). Yet, the mechanisms of Hg methylation, and particularly molecular and cellular pathways at the cell level, are not fully understood. Important advances have been made with genetic studies on *Desulfovibrio desulfuricans* ND132 and *Geobacter sulfurreducens* PCA strains demonstrating that *hgcAB* genes were required for mercury methylation, and that Hg methylation would thus occur in the cytosol, at the cytoplasmic membrane level (Parks et al., 2013; An et al., 2019). Nevertheless, *hgcAB* genes and their expression do not explain the differences in MeHg production under different metabolic conditions or between different methylators, thus suggesting that other parameters including both environmental and physiological ones are involved in the process (Gilmour et al., 2013; Goñi-Urriza et al., 2015).

The uptake of Hg by bacteria is still controversial between passive and active Hg uptake. Schaefer et al. (Schaefer and Morel, 2009; Schaefer et al., 2011, 2014) have proposed an energy-dependent active process where Hg entered the cell using Zn transporters. However, passive uptake of neutral species of inorganic mercury such as HgS nanoparticles, Hg-thiol_2_ [Hg(SR)_2_], HgCl_2_ and aqueous HgS° (HgS_aq_) was also reported (Benoit et al., 2001; Hsu-Kim et al., 2013). Recently An et al. (2019) demonstrated that active transport was not required for IHg uptake in ND132. They also suggested that the uptake could be mediated by thiol-containing membrane proteins and that adsorption would be a first step in the methylation process (An et al., 2019). These ligand-exchange reactions at the cell surface have been considered as a key step in the uptake of Hg for both methylating (An et al., 2019), and non-methylating strains (Mishra et al., 2017; Thomas et al., 2018, 2019). However, the role of the cell surface thiols in Hg methylation has been recently discarded by reporting that the blocking of these surface ligands did not decrease Hg methylation (Thomas et al., 2020). The authors suggested that the coordination of Hg with S ligands in the cell could influence MeHg production (Thomas et al., 2020). Produced MeHg would be exported by the cells (Pedrero et al., 2012; Lin et al., 2015; Liu et al., 2016; Qian et al., 2018), but the exported MeHg chemical forms are still unknown.

The counterpart process, demethylation of MeHg, has been much less studied whereas the net MeHg concentration in the environment results from both processes (Bridou et al., 2011). It can be achieved abiotically or biotically, and two mechanisms can be performed by microorganisms: the well-known reductive demethylation, mediated by aerobic bacteria harboring *mer* operon (Barkay et al., 2003), and the oxidative MeHg demethylation by anaerobic bacteria including sulfate-reducing bacteria, methanogens and methanotrophs (Oremland et al., 1991; Lu et al., 2017) which is mostly unknown. Reductive demethylation occurs at high concentration of Hg in contrast to oxidative demethylation (Barkay et al., 2003), indicating that MeHg demethylation process depends on Hg levels.

In this context, we investigated the effect of Hg concentration on mercury methylation and demethylation in two SRB strains, *Pseudodesulfovibrio hydrargyri* BerOc1 that both methylates and demethylates mercury, and the non-methylating *Desulfovibrio alaskensis* G200 that only demethylates MeHg. We explored Hg species and cellular localization in bacterial cells to constrain the understanding of Hg transformations. For that, we employed High Energy Resolution Fluorescence Detected – X-ray Absorption Near Edge Structure Spectroscopy (HERFD-XANES) to speciate Hg ligands and synchrotron nano X-ray fluorescence (nano-XRF) and transmission electron microscopy combined to X-ray energy dispersive spectroscopy (TEM-X-EDS) to locate Hg. HERFD-XANES has been proved highly sensitive to probe Hg species (Manceau et al., 2015b; Proux et al., 2017; Thomas et al., 2019, 2020) and we specifically questioned the forms of IHg and MeHg in the two bacteria. We hypothesized that Hg speciation, particularly coordination environment, was affected by Hg concentration and was related to the ability to methylate Hg.

## Materials and Methods

### Culture Conditions and Hg Exposures

Two sulfate reducing bacterial strains have been investigated, *Pseudodesulfovibrio hydrargyri* BerOc1 (formerly *Desulfovibrio sp.* BerOc1) able to both methylate inorganic mercury and to demethylate methylmercury (Bridou et al., 2011; Pedrero et al., 2012), and *Desulfovibrio alaskensis* G200, only able to demethylate MeHg (Pedrero et al., 2012). All growth and assays were made in anaerobic conditions, using vessels cleaned by ultrasonication in successive baths of 10% (V/V) HNO_3_ and HCl and rinsed in ultrapure water. Strains were first grown in the dark at 30°C and pH 7.0–7.1 in a multipurpose medium (MM) under sulfate reducing conditions (SR) with lactate/sulfate as electron donor/acceptor (see Supplementary Data 1). Mid-log phase SR cultures were used to inoculate (10%) MM medium with pyruvate/fumarate (PF condition, S1) instead of lactate/sulfate to limit sulfide production. Cultures in late-log growth phase (maximal optical density (OD 600 nm) ∼ 0.2 or ∼ 10^7^ cell/mL) were washed twice (4000 g, 20–30 min, 4°C) in the MM PF medium to remove free sulfides. A fresh MM PF medium was inoculated with washed cells at 20% to obtain non-sulfidogenic conditions. The medium was reduced with titanium citrate (0.05 mL/L). Cell growth was monitored by optical density measured with a spectrophotometer at 600 nm. Cell counting was performed by flow cytometry using a BD Accuri C6 analyzer (TBMCore). A total of 1.6 mL from each culture was sampled and stored at −80°C in 5% (v/v) filtered formaldehyde. Cells were tagged with 10X SYBR^®^ (Invitrogen) following manufacturer’s instructions. Correlation between optical density and cell abundance was performed to normalize methylmercury and inorganic mercury production assays.

Hg methylation/demethylation experiments were carried out in acid pre-cleaned glass material with PTFE stoppers, using the MM PF medium and washed inoculum, in the dark at 30°C and pH 7.1. To evaluate the effect of Hg concentrations on BerOc1 and G200 growth, on the Hg methylation/demethylation capacities and on the Hg species involved, cultures were spiked either with 0, 0.5, 5 and 50 μM HgCl_2_ (IHg) or with 0, 0.5, 5, and 50 μM CH_3_HgCl (MeHg) at mid-log phase growth and incubated during 24 h. BerOc1 was also exposed to 0.05 μM HgCl_2_. Experiments were done in triplicates. Sulfides were quantified at the end of incubation using the Cline method (Cline, 1969) with a spectrophotometer at 670 nm and concentrations were below the detection limit (∼ 1 μM).

### IHg and MeHg Measurements by GC-ICPMS and Partitioning

IHg and MeHg concentrations in the bulk culture were determined at the end of incubation (24 h) by taking 1 mL of the incubation medium and stopping Hg reactions with addition of 1 mL of HNO_3_ 6N. MeHg and IHg production was expressed as μmol/cell by dividing the quantity of MeHg or IHg measured at the end of the culture (in μmol) by the number of cells measured at the end of the culture.

To determine IHg and MeHg partitioning, the remaining culture was centrifuged (15 min, 10000 *g*, +4°C) to separate the supernatant and the pellet. 1 mL of supernatant was collected, and diluted with 50% (V/V) of HNO_3_ 6N for measurements of IHg and MeHg concentrations. The centrifuged pellet was resuspended in 1 mL of MM PF medium and 100 μL was collected and diluted with 50% (V/V) of HNO_3_ 6N for IHg and MeHg analysis.

Concentrations of IHg and MeHg were determined by double spike species-specific isotope dilution analysis. For that, the samples (bulk cultures, supernatant fractions, and pellets) were extracted on a microwave assisted extraction with previously added HNO_3_ 6N (Bridou et al., 2011). Then the extracts were spiked with appropriate amounts of isotopically enriched ^199^IHg standard and ^201^Hg-enriched MeHg standard, for quantification by isotope dilution method (Bridou et al., 2011). The Hg species were propylated using NaBPr_4_ at pH 4 and extracted into iso-octane after shaking the vials manually for 10 min. Finally, the samples were analyzed by Gas Chromatography-ICP-MS [a Trace GC coupled to a X2 series (Thermo Electron)] and the results were mathematically treated applying isotope pattern deconvolution approaches (Rodríguez-González et al., 2007).

IHg and MeHg concentrations were thus measured in each cellular fraction (pellet and supernatant fractions) and converted into quantities in μg. The percentage of each Hg species in each cellular fraction was calculated by dividing the amount of Hg species in the fraction by the sum of the Hg amount measured in all the fractions (Total Hg = MeHg_supernatant_ + IHg_supernatant_ + MeHg_pellet_ + IHg_pellet_). Mass balance between the sum of the fractions and the added Hg amount was evaluated. Recoveries were found to range between 42 and 100% (average 75%) and no relationship was found between the recoveries and Hg concentration, suggesting that Hg did not precipitate as an insoluble form. The low recoveries might result from losses during the incubation period due to sorption of Hg species on the glass wall and poor accuracy in measuring volumes of the collected fractions.

### TEM-X EDS

BerOc1 exposed to 5 μM HgCl_2_ during 24 h and controls (not exposed to Hg) were investigated. After incubation, cells were centrifuged (4000 *g*, 30 min), rinsed in fresh MM PF medium free of Hg, and centrifuged (10000 *g*, 5 min). The pellet was embedded in agarose 4% at 37°C and agarose pieces were mixed with cryo-protectant (bovin serum albumin 20%). High pressure freezing was performed immediately using the EM-HPM 100 Leica microsystem. Freeze-substitution was done using an Automatic Freeze Substitution System AFS2 (Leica microsystem). The frozen bacterial samples were transferred under LN_2_ into cryovials containing 0.2% uranyl acetate in anhydrous acetone. The vials were placed in the AFS2 at −90°C for 3 days and four washings in acetone were done at −55°C. Samples were then immersed in Lowicryl HM20/acetone mixture during 24 h and pure HM20 for 32 h before polymerization with UV during 48 h at −55°C. Samples were warmed to 20°C for 25 h. Embedded samples were then cut in 70 nm thin sections as described in Penen et al. (2017) and deposited on TEM copper grids with carbon film. Sections were imaged with a FEI TECNAI 12 microscope with an accelerating voltage of 120 kV and equipped with an X-Energy Dispersive Spectroscopy (X-Flash 6T 60 Bruker synergie 4). X-EDS fluorescence spectra were collected in nanoprobe mode (50 nm of diameter) in some regions of interest.

### Synchrotron-Based Measurements

#### HERFD-XANES

##### Preparation of Hg reference compounds and bacterial samples

Solid and liquid Hg reference compounds (including IHg and MeHg) were prepared as described in Supplementary Data 2 and analyzed by Hg HERFD-XANES spectroscopy to interpret spectra from experimental bacterial pellets.

At the end of incubations with IHg and MeHg, BerOc1, and G200 cultures were centrifuged (4000 *g*, 30 min), rapidly rinsed once in fresh MM PF medium free of Hg, and twice in ultrapure water. After centrifugation (10000 *g*, 5 min), the bacterial pellet was collected and prepared as a frozen pellet in liquid N_2_ and kept in LN_2_ until measurements. Frozen bacterial pellets and Hg references were then transferred into a helium cryostat operating at 10K for measurements.

##### HERFD-XANES measurements

Hg speciation was investigated using Hg L_3_-edge (12.284 keV) HERFD-XANES spectroscopy on FAME-UHD beamline at ESRF (Grenoble, France). HERFD –XANES spectra were collected by selecting the Hg Lα1 (3d_5/2_ = > 2p_3/2_) fluorescence line using 5 spherically bent Si(111) crystal analyzers aligned in Bragg position (Proux et al., 2017). The diffracted intensity was measured with a Si drift detector (SDD). Calibration of the monochromator was done using a Se foil (maximum of the first derivative set at the Se K-edge position: 12.658 keV). HERFD-XANES data treatment was performed using ATHENA software (Ravel and Newville, 2005). Normalized HERFD-XANES spectra were treated by linear combination fitting (LCF) of Hg reference compounds spectra. The quality of the fit was estimated by the normalized sum-squared residual parameter NSS = Σ(μ_xanes_ – μ_fit_)^2^/Σ(μ_xanes_)^2^ in the energy range 12.272 – 12.362 keV. The precision on the proportion was estimated to 2% (Proux et al., 2017).

#### Cellular Hg Localization by Nano X-Ray Fluorescence

BerOc1 cultures exposed to 0.5, 5, and 50 μM HgCl_2_ and to 5 μM CH_3_HgCl in MM PF medium during 24 h were rapidly rinsed as described for HERFD-XANES measurements. A few μL of suspension were deposited on Si_3_N_4_ membranes, blotted and plunged in liquid ethane using the Leica EM-GP2 plunge freezer. Samples were then slowly freeze-dried during 11 h using various temperature steps from – 120°C to 25°C. Nano-XRF measurements were done at ambient temperature.

Nano-X ray fluorescence was carried out on ID16B at ESRF. The incident beam was used in pink beam mode (energy bandwidth ΔE/E≈10^–2^) at 17.5 keV and the photon flux was 5 × 10^11^ ph/s. It was focused to reach 60 nm (H) × 50 nm (V) lateral resolution on the sample using KB mirrors while two SDD detectors (six elements) positioned at 15° with respect to the sample recorded the fluorescence signal (Martinez-Criado et al., 2016). Fluorescence maps were collected with a 25 nm or 50 nm step size and a 100–400 ms dwell time. XRF spectra were fitted using the PYMCA software (Solé et al., 2007) to obtain elemental maps.

## Results

### Effects of IHg and MeHg Concentrations on Growth and Hg Methylation and Demethylation

**FIGURE 1:**
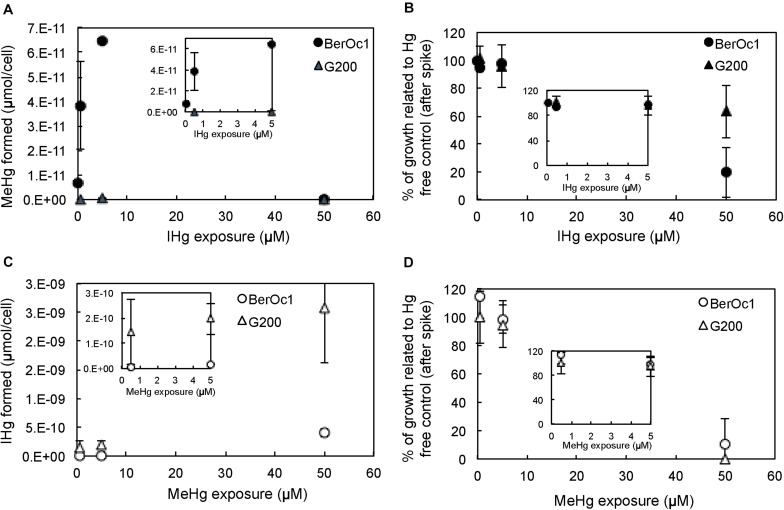
MeHg production by BerOc1 and G200 **(A)**, and their cell growth **(B)**, IHg production by BerOc1 and G200 **(C)** and their cell growth **(D)** depending on IHg **(A,B)** and MeHg **(C,D)** exposure. Error bars correspond to standard deviation calculated on results from two or three replicates. Insets correspond to zooms on the smallest IHg and MeHg exposures (from 0.05 to 5 μM).

The effect of IHg and MeHg concentrations was evaluated by measuring bacterial growth and production of MeHg and IHg. MeHg and IHg production per cell was reported for each strain (Figure 1). For BerOc1 strain, an increase of MeHg production was observed from an IHg exposure of 0.05 μM to 5 μM with a production of MeHg amounting to 6.9 × 10^–12^ μmol/cell at 0.05 μM and to 6.5 × 10^–11^ μmol/cell at 5 μM (Figure 1A). For these IHg exposures, BerOc1 growth was not affected (Figure 1B). A relatively limited increase of MeHg production (a factor 10) compared to the high increase of IHg concentration (a factor 100) was thus observed, suggesting a saturation of Hg methylation. The methylation potentials – calculated as

$$\frac{M\,e\,H\,g\,p\,r\,o\,d\,u\,c\,e\,d}{t\,o\,t\,a\,l\,H\,g}*100$$

– decreased from 24.2% at 0.05 μM IHg to 23.2% at 0.5 μM and to 3.1% at 5 μM IHg. At 50 μM IHg, the methylation of Hg was abolished while growth was strongly impaired (∼ 20% of growth compared to the control), confirming that methylation required intact biological functions. As expected, G200 strain did not methylate IHg, and similarly to BerOc1, its growth was not affected until 5 μM IHg exposure. At 50 μM IHg, G200 growth was also affected (∼60% of growth compared to the control).

For both strains, IHg produced by demethylation increased with increasing concentrations of MeHg in the whole range tested (Figure 1C). The IHg formed at 50 μM MeHg was the highest (6.6 × 10^–11^ μmol/cell for BerOc1 and 9.4 × 10^–10^ μmol/cell for G200) whereas the bacterial growths were highly impaired (Figure 1D), thus indicating that demethylation occurred whatever the physiologic state of cells and did not require an intact biological activity.

### Partitioning of IHg and MeHg

**FIGURE 2:**
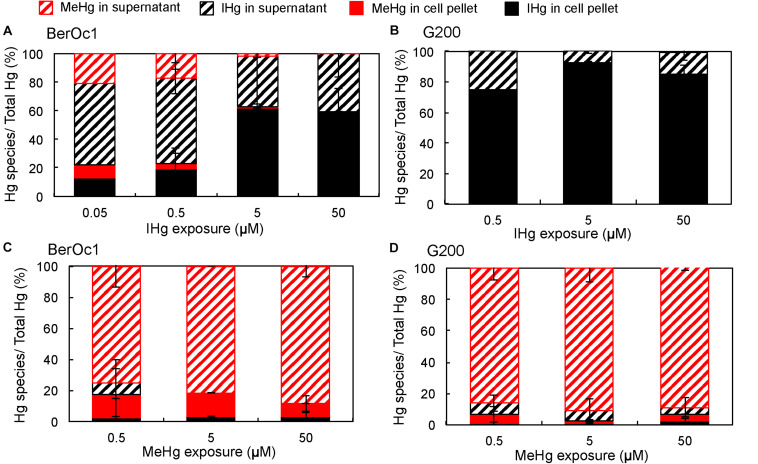
Partitioning of IHg and MeHg (in% of total Hg) in the cell pellet and in the supernatant for BerOc1 **(A,C)** and G200 **(B,D)** exposed to various concentrations of IHg **(A,B)** or MeHg **(B,C)**. Error bars correspond to standard deviation calculated on results from two or three replicates.

The effect of Hg concentrations on the partitioning of IHg and MeHg between the cell-associated fraction and the supernatant after 24 h of incubation was evaluated for both strains. Hg partitioning in BerOc1 depended on IHg concentration (Figure 2A). At the lowest concentrations (0.05 and 0.5 μM) IHg was mainly localized in the supernatant, with proportions ranging between 50 and 60% of total Hg. In contrast, at 5 and 50 μM IHg exposure, IHg was predominantly associated with the cell pellet (>60%) indicating either a high adsorption/internalization of IHg by the cells or the presence of IHg particles in the medium that were retained in the pellet fraction after centrifugation. For 0.05, 0.5, and 5 μM IHg exposures, MeHg produced by BerOc1 was mainly found in the supernatant, suggesting the export of MeHg. For G200, the main part of IHg was encountered in the cell pellet whatever the IHg concentration (Figure 2B). As for BerOc1, it may result from adsorption/internalization of IHg by the cells or from formation of IHg particles in the medium.

Hg partitioning did not differ much between strains and between the various MeHg concentrations of exposure (Figures 2C,D): 75 to 92% of Hg was found as MeHg in the supernatant and the proportion of IHg was low in both supernatant and cell pellet (<10%). Collectively, these results and the increase of the production of IHg per cell with increasing MeHg exposure, support the hypothesis that demethylation is not limited by an intact biological activity contrary to Hg methylation.

### Hg Speciation by Hg L_3_-Edge HERFD-XANES

**FIGURE 3:**
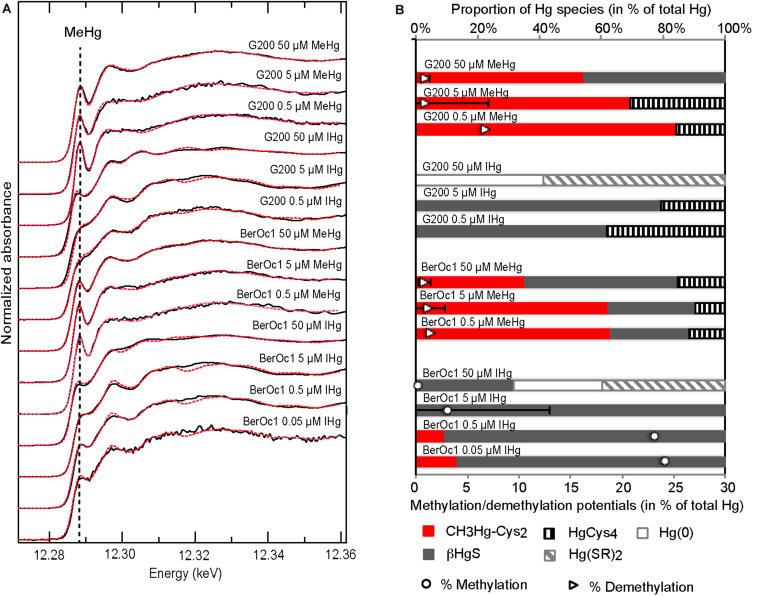
Mercury speciation in BerOc1 and G200 by Hg L_3_-edge HERFD-XANES spectroscopy. **(A)** Spectra of BerOc1 and G200 exposed to various concentrations of MeHg and IHg (black lines) and their linear combination fit (dashed red lines). **(B)** Proportion of Hg species determined from linear combination fitting of the spectra from BerOc1 and G200 displayed in **(A)** plotted with the methylation and demethylation potentials.

High-energy-resolution-fluorescence-detected-X-ray-absorption -near-edge-structure spectroscopy was applied on BerOc1 and G200 pellets to identify the Hg species depending on the concentration of IHg and MeHg exposure (Figure 3). Distinct spectral features in the near edge area from Hg reference compounds (Supplementary Figure 1) attested the very good sensibility of HERFD-XANES spectroscopy, particularly for sulfur-containing ligands and the ability to distinguish linear two coordination [αHgS, Hg-Cysteine_2_, Hg(SR)_2_, Hg-thiol resin] from tetragonal coordination (βHgS, Hg-cysteine_4_). Bacteria exposed to IHg or MeHg had different Hg speciations (Figure 3A). When exposed to MeHg, BerOc1 and G200 had both HERFD-XANES spectra showing a peak at 12.288 keV similarly to methylated Hg references, and the modulation after edge was typical of CH_3_Hg-thiol species (Supplementary Figure 1). In contrast, when exposed to IHg, these spectral signatures vanished, indicating that MeHg was not the predominant species.

In the methylating strain BerOc1, linear combination fits (Figure 3B and Supplementary Table 1) indicated that produced MeHg was under a CH_3_Hg-cysteine_2_ form for the lowest exposures with proportion of 13% for 0.05 μM IHg and 9% for 0.5 μM IHg. No methylated Hg species was detected in the cell pellet for the 5 μM exposure, probably because the proportion of the methylated forms was too low compared to total Hg (methylation potential of 3.1%). Therefore, the proportion of methylated Hg forms in the cell pellet decreased with increasing IHg exposure in agreement with the methylation potentials. The βHgS form was by far the dominant species identified for BerOc1 from 0.05 μM IHg to 5 μM IHg (84, 90, and 100% for 0.05, 0.5, and 5 μM IHg exposures, respectively). The highest IHg exposure (50 μM) showed a different pattern with 31% βHgS, 40% Hg(SR)_2_ and 29% Hg(0). The reliability of this Hg(0) proportion is questionable since the ability of this strain to reduce Hg(II) under growing conditions is very low, near absent (unpublished data). However, the fit agreement was corroborated by the maximum of the derivatives of Hg(0) spectrum and BerOc1 50 μM IHg spectrum, that both displayed a shift to lower energies compared to other Hg references and other IHg exposures (Supplementary Figure 2). Overall, we can infer that Hg speciation was drastically changed compared to lower exposures. For the non-methylating strain G200, obviously, no methylmercury species was identified when exposed to IHg. The βHgS species accounted for more than 61% of the Hg species while a second tetracoordinated Hg, Hg-cysteine_4_ was also identified for 0.5 and 5 μM IHg exposures. Similarly to BerOc1, G200 cells exposed to 50 μM IHg had a different speciation identified as 41% Hg(0) and 58% Hg(SR)_2_.

Hg speciation in BerOc1 exposed to 0.5 and 5 μM MeHg could be described with predominant CH_3_Hg-cysteine_2_ (62%) while the βHgS form and tetracoordinated Hg-cysteine occurred as secondary species [25 and 27% of βHgS and 11 and 10% of Hg-cysteine_4_ for 0.5 and 5 μM, respectively (Figure 3B and Supplementary Table 1)]. The highest MeHg exposure (50 μM) exhibited a different speciation with dominance of βHgS (49%), CH_3_Hg-cysteine_2_ (35%) and Hg-cysteine_4_ (15%). G200 exposed to MeHg showed also spectral pattern with dominance of CH_3_Hg-cysteine_2_ (from 83 to 68% and 54% for 0.5, 5, and 50 μM MeHg, respectively) while Hg-cysteine_4_ was identified as the only secondary phase for the two lowest exposures. βHgS was detected as the secondary species in the G200 for 50 μM MeHg exposure.

### Hg Localization by TEM-X-EDS and Nano-XRF

**FIGURE 4:**
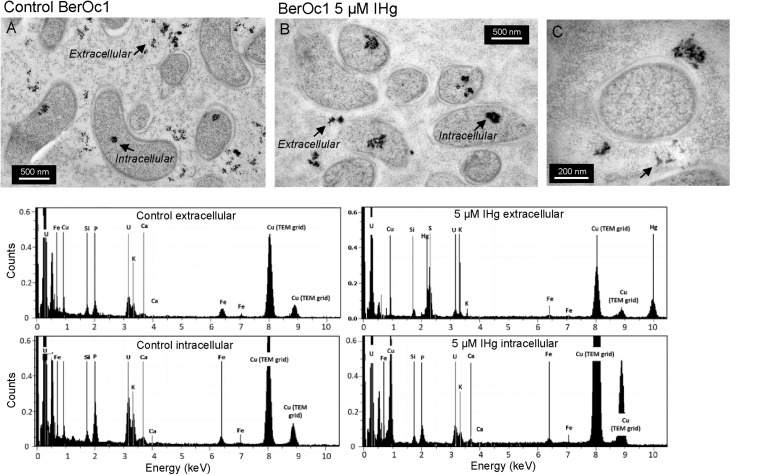
TEM images of thin sections from BerOc1 control **(A)** and exposed to 5 μM IHg **(B,C)** with X-EDS spectra collected on extracellular and intracellular dense aggregates (arrows). Hg was detected in some extracellular nano-particles.

Transmission electron microscopy combined to X-ray energy dispersive spectroscopy and nano-XRF were used to evaluate both the impact of Hg on the cell physiology and to locate Hg at the cell level in BerOc1 strain exclusively. TEM images showed that Hg at 5 μM IHg did not affect BerOc1 cells since control and 5 μM IHg exposed cells had similar size, membranes and periplasmic space and cytoplasm (Figure 4). Intracellular cytoplasmic dense material composed of P, Ca, K, and Fe was evidenced for both control and Hg exposed cells, likely corresponding to calcium iron polyphosphate bodies observed inside microbial cells (Kornberg, 1995; Cosmidis et al., 2014). Nanometer-sized extracellular dense material was present in both cultures (Figure 4 and Supplementary Figure 3). It was composed of P, K, Ca, Fe for the control while in the IHg exposed culture, in addition to these compounds, Hg and S enriched nano-sized aggregates were also detected. No Hg could be detected inside the bacterial cells. We then used nano-XRF to potentially detect a more diluted pool of Hg, due to its better sensitivity compared to X-EDS.

**FIGURE 5:**
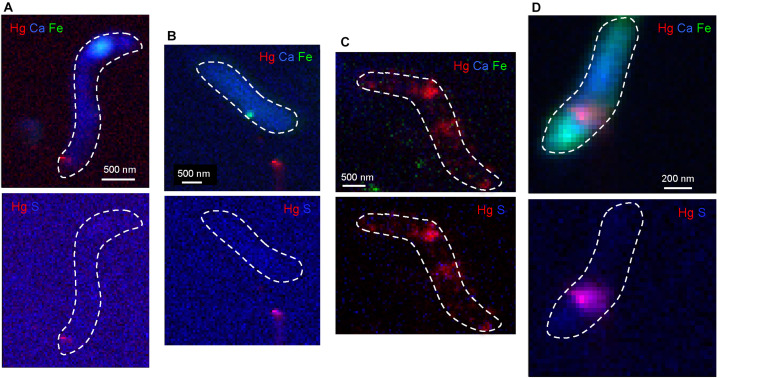
Tricolor (RGB, upper line) nano-XRF maps of the distribution of Hg (red), Fe (Green) and Ca (Blue) and bicolor (RB, lower line) nano-XRF maps of Hg (red) and S (Blue) in microbial cells of BerOc1 exposed to 0.5 μM IHg **(A)**, 5 μM IHg **(B)**, 50 μM IHg **(C)**, and 5 μM MeHg **(D)** during 24 h. Incident energy = 17.5 keV, step size = 50 nm for **(B,C)** and 25 nm for **(A,D)**, counting time = 100 ms/pt for **(B,C)**, and 200 ms/pt for **(A)** and 400 ms/pt for **(D)**. Dashed lines in white delimit the bacterial cells.

Nano-X ray fluorescence maps showed that Hg occurred as nano-sized hot spots located at the interface cell/medium or in the extracellular medium for 0.5 and 5 μM IHg exposures (Figures 5A,B). In these spots, Hg was co-located with S, and could correspond to the βHgS identified by HERFD-XANES. For the highest exposure (50 μM), Hg imaging had a different pattern and Hg was rather associated with the cell with lower S ratio than the other IHg exposures as shown by bicolor Hg/S maps (Figure 5C). Distribution of essential elements (Ca, Fe) for this high exposure and the absence of the intracellular (Fe, Ca)- hot spot that was observed for the lower exposures attested that the cell homeostasis was affected probably due to the toxic level of mercury. It also corresponded to a decrease of cell growth and an odd Hg speciation [composed of βHgS, Hg(0) and Hg(SR)_2_)] identified by HERFD-XANES. When exposed to 5 μM of MeHg (Figure 5D), contrary to IHg exposure, Hg was encountered as more diffused at the BerOc1 cell level and not as nano-sized hot spots in the extracellular medium or at the cell/medium interface, suggesting a different pattern of Hg trafficking in the cell. Hg was still associated with S in agreement with a S-containing binding environment.

## Discussion

### Hg Methylation as a Saturated Process

Our results indicated that MeHg production per cell increased with increasing IHg concentration in the range of 0.05 μM – 5 μM IHg concentrations. The non-linearity between IHg concentrations and MeHg production also suggested that the Hg methylation mechanism tended to be saturated in the highest part of this range of concentrations. Increasing MeHg production has been also observed for *D. desulfuricans* strain ND132 from 5 ppb to 100 ppm Hg (25 nM to 400 μM Hg) under sulfidogenic conditions (Gilmour et al., 2011), and the fraction of Hg converted into MeHg decreased significantly as Hg concentrations increase. In estuarine sediment slurries, King et al. (1999) suggested that the non-linear relationship between MeHg production and IHg concentration was due to saturation of methylating enzymes in SRB, but not clear evidence of the effect of Hg concentration on MeHg production is available. As an intracellular process, it can be inferred that Hg methylation is related to Hg uptake by the bacterial cell, and that cell sorption can be the first step for Hg uptake. The addition of thiols compounds (cysteine, glutathione, penicillamine) can remobilize Hg for uptake and methylation, possibly by acting as competitive ligand exchange with bacterial cell surface sites (Liu et al., 2016). Monitoring Hg methylation with time in non-growing ND132 cells, Liu et al. (2016) observed a Hg methylation plateau after 24 h of incubation with 25 nM HgCl_2_ and suggested that this plateau resulted from immobilization of Hg(II) by strong surface cellular binding (Liu et al., 2016). Recent speciation modeling also pointed out that metal complexation with cell surface ligands should be involved in Hg uptake and Hg methylation (Adediran et al., 2019). However, recently, Thomas et al. (2020) evaluated the role of surface thiol sites by blocking them with a highly efficient thiol binding ligand and did not find any effect in Hg methylation in *G. sulfurreducens* PCA, thus impeding the hypothesis that cell binding with thiols was necessary for this strain. Here, when Hg methylation occurred, both nano-XRF and TEM-X EDS indicated Hg nano-particles in the extracellular medium or at the cell/medium interface that could have been exported out of the cell or formed by biomolecules produced by the cell (see next paragraph). In contrast, at high Hg exposure (50 μM), nano-XRF showed that most of Hg was associated to the cell. This high internalization of Hg, could be due to an altered controlled uptake, but also to an impaired efflux system, thus resulting in an accumulation of Hg in the cell.

### Formation of βHgS Like Compounds

Tetracoordinated βHgS-like compound was identified for both methylating and non-methylating strains and for IHg and MeHg exposures. This phase might correspond to the Hg/S enriched nano-sized extracellular particles observed by TEM-X EDS and nano-XRF although it was beyond the abilities of the imaging techniques to distinguish MeHg from IHg. We do not know how these particles are formed. If they formed at the beginning of the exposure in the culture medium, they should have been available for Hg methylation. Indeed, at 5 μM of IHg, where the maximum of methylation was observed, only βHgS was identified by HERFD-XANES. The availability of HgS_(s)_ particles for Hg methylation by *G. sulfurreducens* PCA has been observed. For instance, when sulfides (from 20 to 500 nM) and Hg(II) (20–25 nM) were added in the culture medium before PCA inoculation, all Hg precipitated as solid HgS but Hg methylation occurred, thus indicating that Hg from HgS was available for methylation (Adediran et al., 2019). The authors and others suggested that size and structure of HgS play a role in Hg bioavailability and Hg methylation by bacteria (Zhang et al., 2012; Pham et al., 2014; Adediran et al., 2019). Other studies confirmed the bioavailability of HgS_(s)_ nanoparticles (Graham et al., 2012; Thomas et al., 2018). Alternatively, the βHgS-like particles observed in our study could be originated from Hg-thiol compounds formed in the cell and exported since IHg associated to biomolecules was identified in the cytosol of BerOc1 and G200 (Pedrero et al., 2012). Finally, another possibility is that they could form due to interactions of S-containing biomolecules produced by the bacteria with Hg at the vicinity of the cell. For instance, Adediran et al. (2019) demonstrated that PCA strain biosynthesized and exported thiols, mainly cysteine, in the culture medium (Adediran et al., 2019). It can be hypothesized that BerOc1 and G200 produce thiols that can interact with Hg, and it is possible that they evolve toward a more crystallized form. Indeed, Manceau et al. proposed the formation of βHgS from Hg(II)-(cysteine ethyl ester)_2_ and from Hg(II)-thiols of natural organic matter through an abiotic way (Manceau et al., 2015a; Enescu et al., 2016). βHgS was also identified in *Escherichia coli* and *G. sulfurreducens* cultures probably due to precipitation with endogenous sulfides sources (Thomas and Gaillard, 2017; Thomas et al., 2018). Interestingly Thomas et al. (2020) pointed out that Hg-S_3_/S_4_ enhanced Hg uptake and methylation in *G. sulfurreducens* PCA whereas Hg-S_2_ was related to low Hg methylation. Here we identified predominant tetracoordinated Hg-S when MeHg was produced in BerOc1 corroborating the important role of Hg ligands and coordination in the mechanism of Hg methylation.

### Methylated Hg Is Also Bound to Thiol Biomolecules

We report the formation of MeHg-cysteine_2_ like molecules by BerOc1 following IHg exposure. Since Hg methylation is expected to occur intracellularly and MeHg was mainly encountered in the supernatant in our study, the methylating strain might export the produced MeHg-thiol. The MeHg export has been proposed for BerOc1 (Pedrero et al., 2012) and other strains such as *D. desulfuricans* ND132 and *G. sulfurreducens* PCA (Schaefer and Morel, 2009; Schaefer et al., 2011; Lin et al., 2015; Liu et al., 2016). Pedrero et al. (2012) evidenced biomolecules of high molecular weight (>70, 20, and 17 kDa) binding MeHg in the extracellular medium of BerOc1 but the chemical nature of these molecules remained unidentified. We also evidenced by Hg partitioning that part of MeHg (2–16%) for both methylating and non-methylating strains exposed to MeHg was found at the end of the growth in the pellet and occurred as MeHg-thiols (MeHg-cysteine_2_ like form) based on HERFD-XANES. However, we could not state if the Hg present at the cell level and observed by nano-XRF corresponded to this methylated form.

### Hg Demethylation as a Non-saturated Process

It was shown here that for both strains, the production of IHg was the highest for the highest MeHg concentrations. Similar conclusion was reported for various sulfate-reducing bacteria exposed to lower MeHg concentrations (Bridou et al., 2011). Some methanotrophs also increased their ability to demethylate MeHg with increasing MeHg concentrations (Lu et al., 2017). Taken together, these results support that demethylation is not limited by an intact biological activity. MeHg demethylation might nevertheless require interaction with the cell since Hg associated to the cell was detected by nano-XRF. Here, between 2 and 16% of total Hg was present as MeHg (identified as MeHg-Cysteine_2_ by HERFD-XANES) in the pellet whatever the strain and the MeHg initial concentration. The IHg formed by demethylation in the pellet was identified as tetracoordinated Hg species modeled by both βHgS and Hg-(cysteine)_4_, again pointing out that this Hg coordination is of importance in cell Hg pathway. We note that the proportion of inorganic Hg species identified by HERFD-XANES in the pellet – ranging from 15 to 64% – is high compared to Hg partitioning (IHg in the pellet accounts for 10–38% of the total Hg in the pellet). Although, it is difficult to compare these results since the bacterial pellet was washed before XANES measurement in contrast to partitioning, both methods indicated an increase of IHg with increasing MeHg exposure.

This study thus suggests the saturation of Hg methylation process with Hg concentrations in contrast to MeHg demethylation. It also highlights the predominance of tetracoordinated inorganic Hg-S species in Hg intracellular processes, and corroborates its role in Hg methylation with important environmental implications. This speciation should be further investigated in future environmental studies.

### Supporting Information

Composition of the culture medium, strains, preparation of Hg references for HERFD-XANES, Hg L_3_-edge HERFD-XANES reference spectra, derivatives of HERFD-XANES bacterial spectra and Hg references spectra, TEM-X EDS analyses, proportion of Hg species determined by LCF.

## Data Availability Statement

The original contributions presented in the study are included in the article/supplementary material, further inquiries can be directed to the corresponding author.

## Author Contributions

M-PI, MM, and MG-U designed the experiments, participated to the measurements, data treatment/discussion, and wrote the manuscript. MA did the experiments/measurements and was involved in data treatment. IK was involved in XANES measurements. RT was involved in nano-XRF measurements. EG and MP were involved in TEM preparations and measurements. ET was involved in GC-ICPMS measurements. All authors contributed to the article and approved the submitted version.

## Conflict of Interest

The authors declare that the research was conducted in the absence of any commercial or financial relationships that could be construed as a potential conflict of interest.

## References

[B1] Adediran G. A. Liem-Nguyen V. Song Y. Schaefer J. K. Skyllberg U. Björn E. (2019). Microbial biosynthesis of thiol compounds: implications for speciation, cellular uptake, and methylation of Hg(II). *Environ. Sci. Technol.* 53 8187–8196. 10.1021/acs.est.9b01502 31257868

[B2] An J. Zhang L. Lu X. Pelletier D. A. Pierce E. M. Johs A. (2019). Mercury Uptake by Desulfovibrio desulfuricans ND132: passive or active? *Environ. Sci. Technol.* 53 6264–6272. 10.1021/acs.est.9b00047 31075193

[B3] Azaroff A. Goñi Urriza M. Gassie C. Monperrus M. Guyoneaud R. (2020). Marine mercury-methylating microbial communities from coastal to Capbreton Canyon sediments (North Atlantic Ocean). *Environ. Pollut.* 262:114333. 10.1016/j.envpol.2020.114333 32443198

[B4] Barkay T. Miller S. M. Summers A. O. (2003). Bacterial mercury resistance from atoms to ecosystems. *FEMS Microbiol. Rev.* 27 355–384. 10.1016/s0168-6445(03)00046-9 12829275

[B5] Benoit J. M. Gilmour C. C. Mason R. P. (2001). Aspects of bioavailability of mercury for methylation in pure cultures of Desulfobulbus propionicus (1pr3). *Appl. Environ. Microbiol.* 67 51–58. 10.1128/aem.67.1.51-58.2001 11133427 PMC92513

[B6] Bridou R. Monperrus M. Gonzalez P. R. Guyoneaud R. Amouroux D. (2011). Simultaneous determination of mercury methylation and demethylation capacities of various sulfate-reducing bacteria using species-specific isotopic tracers. *Environ. Toxicol. Chem.* 30 337–344. 10.1002/etc.395 21038431

[B7] Cline J. D. (1969). Spectrophotometric determination of hydrogen sulfide in natural waters. *Limnol. Oceanogr.* 14 454–458. 10.4319/lo.1969.14.3.0454

[B8] Cosmidis J. Benzerara K. Morin G. Busigny V. Lebeau O. Jézéquel D. (2014). Biomineralization of iron-phosphates in the water column of Lake Pavin (Massif Central. France). *Geochim. Cosmochim. Acta* 126 78–96. 10.1016/j.gca.2013.10.037

[B9] Enescu M. Nagy K. L. Manceau A. (2016). Nucleation of mercury sulfide by dealkylation. *Sci. Rep.* 6:39359. 10.1038/srep39359 PMC5171843 27991599

[B10] Gilmour C. C. Elias D. A. Kucken A. M. Brown S. D. Palumbo A. V. Schadt C. W. (2011). Sulfate-reducing bacterium *Desulfovibrio desulfuricans* ND132 as a model for understanding bacterial mercury methylation. *Appl. Environ. Microbiol.* 77 3938–3951. 10.1128/aem.02993-10 21515733 PMC3131654

[B11] Gilmour C. C. Podar M. Bullock A. L. Graham A. M. Brown S. D. Somenahally A. C. (2013). Mercury methylation by novel microorganisms from new environments. *Environ. Sci. Technol.* 47 11810–11820. 10.1021/es403075t 24024607

[B12] Gionfriddo C. M. Tate M. T. Wick R. R. Schultz M. B. Zemla A. Thelen M. P. (2016). Microbial mercury methylation in Antarctic sea ice. *Nat. Microbiol.* 1:16127. 10.1038/nmicrobiol.2016.127 27670112

[B13] Goñi-Urriza M. Corsellis Y. Lanceleur L. Tessier E. Gury J. Monperrus M. (2015). Relationships between bacterial energetic metabolism, mercury methylation potential, and hgcA/hgcB gene expression in *Desulfovibrio dechloroacetivorans* BerOc1. *Environ. Sci. Pollut. Res.* 22 13764–13771. 10.1007/s11356-015-4273-5 25772867

[B14] Graham A. M. Aiken G. R. Gilmour C. C. (2012). Dissolved organic matter enhances microbial mercury methylation under sulfidic conditions. *Environ. Sci. Technol.* 46 2715–2723. 10.1021/es203658f 22309093

[B15] Hsu-Kim H. Kucharzyk K. H. Zhang T. Deshusses M. A. (2013). Mechanisms regulating mercury bioavailability for methylating microorganisms in the aquatic environment: a critical review. *Environ. Sci. Technol.* 47 2441–2456. 10.1021/es304370g 23384298

[B16] Jones D. S. Walker G. M. Johnson N. W. Mitchell C. P. J. Coleman Wasik J. K. Bailey J. V. (2019). Molecular evidence for novel mercury methylating microorganisms in sulfate-impacted lakes. *ISME J.* 13 1659–1675. 10.1038/s41396-019-0376-1 30809010 PMC6776050

[B17] King J. K. Saunders F. M. Lee R. F. Jahnke R. A. (1999). Coupling mercury methylation rates to sulfate reduction rates in marine sediments. *Environ. Toxicol. Chem.* 18 1362–1369. 10.1002/etc.5620180704

[B18] Kornberg A. (1995). Inorganic polyphosphate: toward making a forgotten polymer unforgettable. *J. Bacteriol.* 177 491–496. 10.1128/jb.177.3.491-496.1995 7836277 PMC176618

[B19] Lin H. Lu X. Liang L. Gu B. (2015). Thiol-facilitated cell export and desorption of methylmercury by anaerobic bacteria. *Environ. Sci. Technol. Lett.* 2 292–296. 10.1021/acs.estlett.5b00209

[B20] Liu Y. R. Lu X. Zhao L. An J. He J. Z. Pierce E. M. (2016). Effects of cellular sorption on mercury bioavailability and methylmercury production by desulfovibrio desulfuricans ND132. *Environ. Sci. Technol.* 50 13335–13341. 10.1021/acs.est.6b04041 27993064

[B21] Lu X. Gu W. Zhao L. Ul Haque M. F. Dispirito A. A. Semrau J. D. (2017). Methylmercury uptake and degradation by methanotrophs. *Sci. Adv.* 3:e1700041. 10.1126/sciadv.1700041 28580426 PMC5451197

[B22] Manceau A. Lemouchi C. Enescu M. Gaillot A. C. Lanson M. Magnin V. (2015a). Formation of Mercury Sulfide from Hg(II)-Thiolate Complexes in Natural Organic Matter. *Environ. Sci. Technol.* 49 9787–9796. 10.1021/acs.est.5b02522 26168020

[B23] Manceau A. Lemouchi C. Rovezzi M. Lanson M. Glatzel P. Nagy K. L. (2015b). Structure, Bonding, and Stability of Mercury Complexes with Thiolate and Thioether Ligands from High-Resolution XANES Spectroscopy and First-Principles Calculations. *Inorganic Chem.* 54 11776–11791. 10.1021/acs.inorgchem.5b01932 26651871

[B24] Martinez-Criado G. Villanova J. Tucoulou R. Salomon D. Suuronen J. P. Laboure S. (2016). ID16B: a hard X-ray nanoprobe beamline at the ESRF for nano-analysis. *J. Synchrotron Radiat.* 23 344–352. 10.1107/s1600577515019839 26698084 PMC5297598

[B25] Mishra B. Shoenfelt E. Yu Q. Yee N. Fein J. B. Myneni S. C. B. (2017). Stoichiometry of mercury-thiol complexes on bacterial cell envelopes. *Chem. Geol.* 464 137–146. 10.1016/j.chemgeo.2017.02.015

[B26] Oremland R. S. Culbertson C. W. Winfrey M. R. (1991). Methylmercury decomposition in sediments and bacterial cultures: involvement of methanogens and sulfate reducers in oxidative demethylation. *Appl. Environ. Microbiol.* 57 130–137. 10.1128/aem.57.1.130-137.1991 16348388 PMC182673

[B27] Parks J. M. Johs A. Podar M. Bridou R. Hurt R. A. Jr. Smith S. D. (2013). The genetic basis for bacterial mercury methylation. *Science* 339 1332–1335. 10.1126/science.1230667 23393089

[B28] Pedrero Z. Bridou R. Mounicou S. Guyoneaud R. Monperrus M. Amouroux D. (2012). Transformation, localization, and biomolecular binding of Hg species at subcellular level in methylating and nonmethylating sulfate-reducing bacteria. *Environ. Sci. Technol.* 46 11744–11751. 10.1021/es302412q 23050725

[B29] Penen F. Isaure M. P. Dobritzsch D. Bertalan I. Castillo-Michel H. Proux O. (2017). Pools of cadmium in *Chlamydomonas reinhardtii* revealed by chemical imaging and XAS spectroscopy. *Metallomics* 9 910–923. 10.1039/c7mt00029d 28598481

[B30] Pham A. L. T. Morris A. Zhang T. Ticknor J. Levard C. Hsu-Kim H. (2014). Precipitation of nanoscale mercuric sulfides in the presence of natural organic matter: structural properties, aggregation, and biotransformation. *Geochim. Cosmochim. Acta* 133 204–215. 10.1016/j.gca.2014.02.027

[B31] Proux O. Lahera E. Del Net W. Kieffer I. Rovezzi M. Testemale D. (2017). High-energy resolution fluorescence detected X-ray absorption spectroscopy: a powerful new structural tool in environmental biogeochemistry sciences. *J. Environ. Qual.* 46 1146–1157. 10.2134/jeq2017.01.0023 29293835

[B32] Qian C. Chen H. Johs A. Lu X. An J. Pierce E. M. (2018). Quantitative proteomic analysis of biological processes and responses of the bacterium *Desulfovibrio desulfuricans* ND132 upon deletion of its mercury methylation genes. *Proteomics* 18:1700479. 10.1002/pmic.201700479 30009483

[B33] Ravel B. Newville M. (2005). ATHENA, ARTEMIS, HEPHAESTUS: data analysis for X-ray absorption spectroscopy using IFEFFIT. *J. Synchrotron Radiat.* 12 537–541. 10.1107/s0909049505012719 15968136

[B34] Rodríguez-González P. Monperrus M. García Alonso J. I. Amouroux D. Donard O. F. X. (2007). Comparison of different numerical approaches for multiple spiking species-specific isotope dilution analysis exemplified by the determination of butyltin species in sediments. *J. Anal. Atomic Spectr.* 22 1373–1382. 10.1039/b706542f

[B35] Schaefer J. K. Morel F. M. M. (2009). High methylation rates of mercury bound to cysteine by *Geobacter sulfurreducens*. *Nat. Geosci.* 2 123–126. 10.1038/ngeo412

[B36] Schaefer J. K. Rocks S. S. Zheng W. Liang L. Gu B. Morel F. M. M. (2011). Active transport, substrate specificity, and methylation of Hg(II) in anaerobic bacteria. *Proc. Natl. Acad. Sci. U.S.A.* 108 8714–8719. 10.1073/pnas.1105781108 21555571 PMC3102380

[B37] Schaefer J. K. Szczuka A. Morel F. M. M. (2014). Effect of divalent metals on Hg(II) uptake and methylation by bacteria. *Environ. Sci. Technol.* 48 3007–3013. 10.1021/es405215v 24512453

[B38] Solé V. A. Papillon E. Cotte M. Walter P. Susini J. (2007). A multiplatform code for the analysis of energy-dispersive X-ray fluorescence spectra. *Spectrochim. Acta Part B Atomic Spectr.* 62 63–68. 10.1016/j.sab.2006.12.002

[B39] Thomas S. A. Catty P. Hazemann J. L. Michaud-Soret I. Gaillard J. F. (2019). The role of cysteine and sulfide in the interplay between microbial Hg(ii) uptake and sulfur metabolism. *Metallomics* 11 1219–1229. 10.1039/c9mt00077a 31143907

[B40] Thomas S. A. Gaillard J. F. (2017). Cysteine Addition Promotes Sulfide Production and 4-Fold Hg(II)-S Coordination in Actively Metabolizing *Escherichia coli*. *Environ. Sci. Technol.* 51 4642–4651. 10.1021/acs.est.6b06400 28353340

[B41] Thomas S. A. Mishra B. Myneni S. C. B. (2020). Cellular mercury coordination environment, and not cell surface ligands, influence bacterial methylmercury production. *Environ. Sci. Technol.* 54 3960–3968. 10.1021/acs.est.9b05915 32097551

[B42] Thomas S. A. Rodby K. E. Roth E. W. Wu J. Gaillard J. F. (2018). Spectroscopic and Microscopic Evidence of Biomediated HgS Species Formation from Hg(II)-Cysteine Complexes: implications for Hg(II) Bioavailability. *Environ. Sci. Technol.* 52 10030–10039. 10.1021/acs.est.8b01305 30078312

[B43] Villar E. Cabrol L. Heimbürger-Boavida L. E. (2020). Widespread microbial mercury methylation genes in the global ocean. *Environ. Microbiol. Rep.* 12 277–287. 10.1111/1758-2229.12829 32090489

[B44] Zhang T. Kim B. Levard C. Reinsch B. C. Lowry G. V. Deshusses M. A. (2012). Methylation of mercury by bacteria exposed to dissolved, nanoparticulate, and microparticulate mercuric sulfides. *Environ. Sci. Technol.* 46 6950–6958. 10.1021/es203181m 22145980

