# Supplementary material for: Relationship Between Hg Speciation and Hg Methylation/Demethylation Processes in the Sulfate-Reducing Bacterium Pseudodesulfovibrio hydrargyri: Evidences From HERFD-XANES and Nano-XRF
Source: Front Microbiol. 2020 Oct 14;11:584715. doi: 10.3389/fmicb.2020.584715 (PMC7591507; doi:10.3389/fmicb.2020.584715)
Supplement: Supplementary file 1 [file Data_Sheet_1.docx]

***Supplementary Material***

**Supplementary Data 1: Growth medium composition, strains and number of cells by flow cytometry**

MM medium: NaCl 171 mM for BerOc1 and 51.3 mM for G200, KCl 6.7 mM, NH_4_Cl, 4.7 mM, MgCl_2_ 2 mM, KH_2_PO_4_ 1.5 mM, CaCl_2_ 0.7 mM, H_3_BO_3_ 4.85 µM, CoCl_2_ 0.79 µM, MnCl_2_ 0.25 µM, ZnCl_2_ 0.30 µM, NiCl_2_ 0.10 µM, Na_2_MoO_4_ 0.07 µM, CuCl_2_ 0.01 µM, FeSO_4_ 3.95 µM, selenite 5.5 µM, tungstate 9.1 µM, vitamins D-biotine 0.03 µM, B3 0.65 µM, B1 0.12 µM, H1 0.29 µM, B5 0.08 µM, B6 0.97 µM, B12 0.06 µM, Tris 10 mM, yeast extract 0.05g/L, pH 7.0.

Under sulfate respiration i.e. sulfate reduction (LS): lactate 20 mM, sulfate 20 mM.

Under fumarate respiration (PF): pyruvate 40 mM, fumarate 40 mM. Under this condition, sulfur is limited and explains low growth.

- Strain BerOc1 (*Pseudodesulfovibrio hydrargyri*), is an incomplete acetate oxidizer isolated from brackish lagoon sediments (Etang de Berre, France), (Ranchou-Peyruse *et al.*, 2018) and has been previously tested for its capacity to methylate Hg and demethylate MMHg (Ranchou-Peyruse *et al.*, 2009; Bridou *et al.*, 2011; Pedrero *et al.*, 2012). This halophilic strain is closely related to the mercury methylating *Desulfovibrio desulfuricans* strain ND132, model microorganism to study mercury methylation (Gilmour *et al.*, 2011; Goñi-Urriza *et al.*, 2020).

- Strain G200 *D*. *desulfuricans* strain G200 was isolated from an oil well souring site (Weimer *et al.*, 1988) and is a *Desulfovibrio* model microorganism that was intensely involved in the development of molecular genetic techniques for this genus over the last 20 years (Rapp & Wall, 1987; Hauser *et al.*, 2011). *Desulfovibrio* *desulfuricans* Strain G200 genome sequencing and annotation made available genomic data that will be valuable to understand mercury methylation by sulfate-reducing microorganisms (Oak Ridge National Laboratory, USA, <http://genome.ornl.gov/microbial/ddes/.html>). In this study, Strain G200 was used as a negative Hg methylating control and was grown in the same medium and conditions as described for Strain BerOc1 unless for salinity that was kept at 51.3 mM instead of 171 mM.

**Supplementary Data 2: Preparation of Hg references for HERFD-XANES**

Powdered αHgS, βHgS, HgO, Hg-acetate, HgCl_2_, and CH_3_HgCl were purchased, diluted in boron nitride (0.8% Hg) and prepared as 5 mm diameter pressed pellets. Solid CH_3_Hg-carboxyl was prepared by adding 12.5 mL of 1mM CH_3_HgCl to 1g BioRex Dow RCOOH resin as described in Skyllberg et al. (Skyllberg *et al.*, 2006). Suspensions were stirred at 200 rpm for 24h, rinsed with ultra pure water and freeze-dried. Solid Hg-thiol and CH_3_Hg-thiols were prepared with the GT74 Dow RSH resin using the same protocol. All resin samples were then prepared as pressed pellets with boron nitride. Thiolate complexes namely linear Hg(SR)_2_ (Hg(SC_6_H_11_)_2_) was prepared according to Alsina et al. (Alsina *et al.*, 1992).

Liquid references, namely Hg-cysteine ([Hg]= 18 mM, M/L= 1/ 2, pH= 2.2, HgCys2), CH_3_HgOH ([Hg]= 43 mM, pH= 9.4), CH_3_Hg-cysteine ([Hg]= 21 mM, M/L = 1 /2, pH= 7.2, CH_3_Hg-Cys2), and CH_3_Hg-glycine ([Hg]= 10 mM, M/L = 1/1, pH= 7.1) were prepared in a glove bag under N_2_ gas and adjusting pH with KOH and HCl. Just before measurements, solutions were mixed with 20% glycerol to prevent ice formation during cooling. Hg(0) was mounted as liquid sample. Hg-cysteine4 spectrum (HgCys4), collected on the same beamline in similar conditions, was kindly provided by Sara Thomas (DOI 10.26302/SSHADE/EXPERIMENT_ST_20161124_001 (Thomas *et al.*, 2019) ).


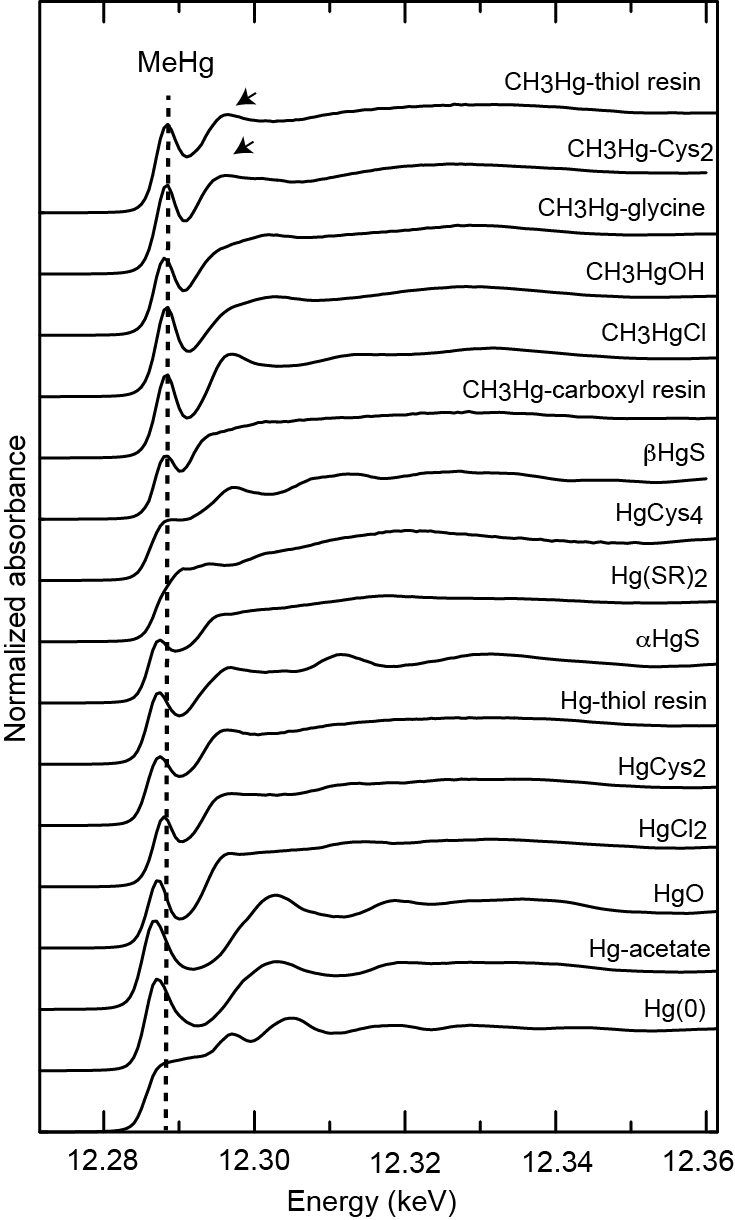


**Supplementary Figure 1:** Hg L_3_-edge HERFD-XANES spectra of Hg reference compounds including methylmercury and inorganic forms. The dashed line indicates the peak present in MeHg species. The arrows indicate the modulation after edge present in MeHg-thiol species. Tetra-coordinated mercury such as metacinnabar (βHgS) and Hg-cysteine_4_ exhibited a flat near-edge whereas linearly coordinated mercury bound to sulfur such as cinnabar (αHgS), Hg-cysteine_2_, Hg(SR)_2_ and Hg-thiol resin had a clear peak shifted to higher energy value compared to mercury bound to oxygen (Hg-acetate, HgO) or chloride. All spectra for methylated species showed a peak at 12.288 keV and modulations beyond the absorption edge then differed with various forms of methylmercury: CH_3_Hg-cysteine_2_ and CH_3_Hg-thiol resin, considered as a proxy for CH_3_Hg-thiol_2_, could be distinguished from the methylated non thiolated species.


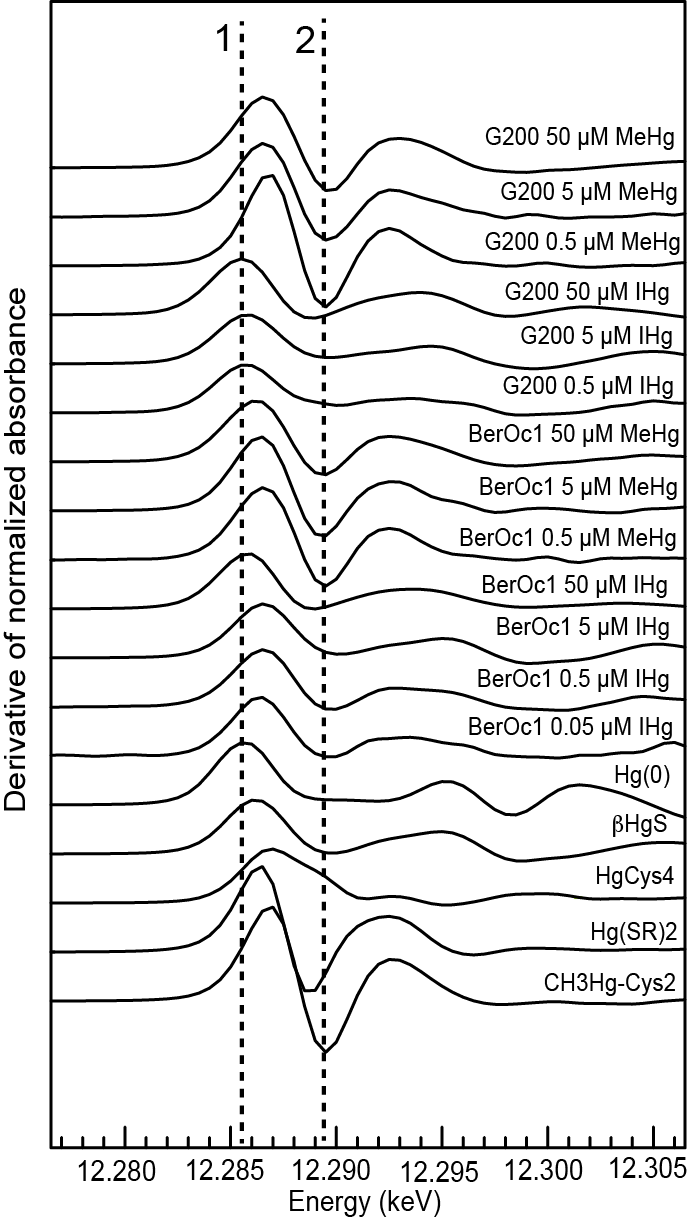


**Supplementary Figure 2:** Derivatives of BerOc1 and G200 Hg L_3_-edge HERFD-XANES spectra and of Hg references used in the LCF. Dashed line 1 indicates the maximum of the derivative for Hg(0) spectrum and dashed line 2 indicates the minimum of the derivative for CH_3_Hg-Cys_2_.


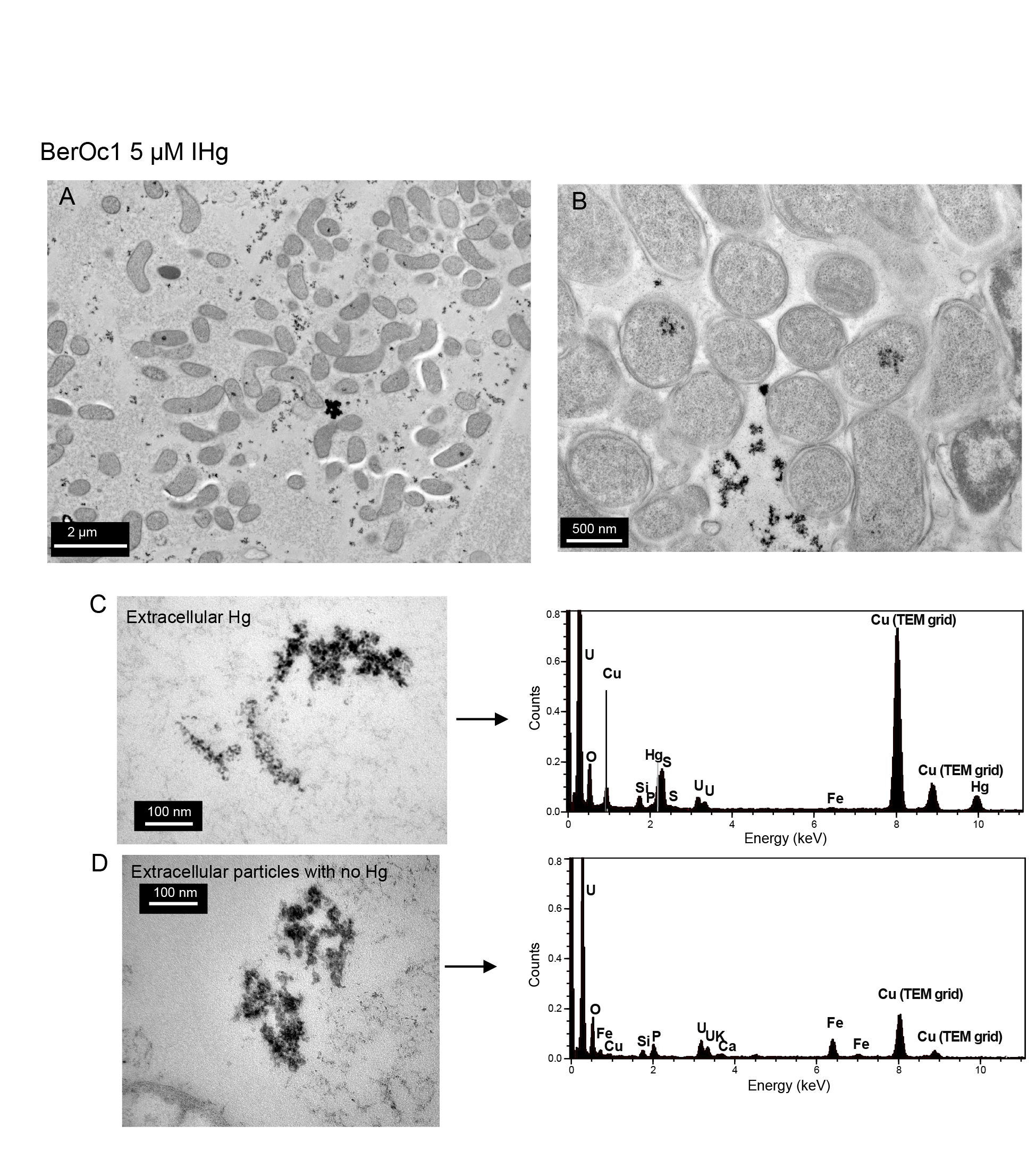


**Supplementary Figure 3:** TEM images of thin sections from BerOc1 exposed to 5 µM IHg (A, B), with X-EDS spectra collected on extracellular particles (C, D). Some of the extracellular nano-scaled particles present in the extracellular medium are composed of Hg/S (C) and others are composed of P, K, Ca and Fe (D, similarly to the control).

**Supplementary Table 1:** Proportion of Hg species (%) determined by linear conbination fitting (LCF) of the Hg L_3_ HERFD-XANES spectra.

|  | βHgS | CH_3_Hg-Cys_2_ | HgCys_4_ | Hg(SR)_2_ | Hg(0) | *NSS* |
| --- | --- | --- | --- | --- | --- | --- |
| G200 50 µM MeHg | 45 | 54 |  |  |  | *2.1 x 10^-5^* |
| G200 5 µM MeHg |  | 68 | 30 |  |  | *3.1 x 10^-4^* |
| G200 0.5 µM MeHg |  | 83 | 15 |  |  | *1.9 x 10^-4^* |
| G200 50 µM IHg |  |  |  | 58 | 41 | *1.6 x 10^-4^* |
| G200 5 µM IHg | 79 |  | 21 |  |  | *4.3 x 10^-4^* |
| G200 0.5 µM IHg | 61 |  | 37 |  |  | *2.4 x 10^-4^* |
| BerOc1 50 µM MeHg | 49 | 35 | 15 |  |  | *4.4 x 10^-4^* |
| BerOc1 5 µM MeHg | 27 | 62 | 10 |  |  | *1.4 x 10^-4^* |
| BerOc1 0.5 µM MeHg | 25 | 62 | 11 |  |  | *2.7 x 10^-4^* |
| BerOc1 50 µM IHg | 31 |  |  | 40 | 29 | *1.6 x 10^-4^* |
| BerOc1 5 µM IHg | 100 |  |  |  |  | *3.6 x 10^-4^* |
| BerOc1 0.5 µM IHg | 90 | 9 |  |  |  | *3.0 x 10^-4^* |
| BerOc1 0.5 µM IHg | 84 | 13 |  |  |  | *5.1 x 10^-4^* |

Normalized Sum-Squared residual parameter *NSS* = Σ(μ_xanes_ – μ_fit_)^2^/Σ(μ_xanes_)^2^ in the energy range 12.272 – 12.362 keV.

**Alsina T, Clegg W, Fraser KA, Sola J. 1992.** Homoleptic cyclohexanethiolato complexes of mercury(II). *Journal of the Chemical Society, Dalton Transactions*: 1393-1399.

**Bridou R, Monperrus M, Gonzalez PR, Guyoneaud R, Amouroux D. 2011.** Simultaneous determination of mercury methylation and demethylation capacities of various sulfate-reducing bacteria using species-specific isotopic tracers. *Environmental Toxicology and Chemistry* **30**: 337-344.

**Gilmour CC, Elias DA, Kucken AM, Brown SD, Palumbo AV, Schadt CW, Wall JD. 2011.** Sulfate-reducing bacterium Desulfovibrio desulfuricans ND132 as a model for understanding bacterial mercury methylation. *Applied and Environmental Microbiology* **77**: 3938-3951.

**Goñi-Urriza M, Klopp C, Ranchou-Peyruse M, Ranchou-Peyruse A, Monperrus M, Khalfaoui-Hassani B, Guyoneaud R. 2020.** Genome insights of mercury methylation among Desulfovibrio and Pseudodesulfovibrio strains. *Research in Microbiology* **171**: 3-12.

**Hauser LJ, Land ML, Brown SD, Larimer F, Keller KL, Rapp-Giles BJ, Price MN, Lin M, Bruce DC, Detter JC, et al. 2011.** Complete genome sequence and updated annotation of Desulfovibrio alaskensis G20. *Journal of Bacteriology* **193**: 4268-4269.

**Pedrero Z, Bridou R, Mounicou S, Guyoneaud R, Monperrus M, Amouroux D. 2012.** Transformation, localization, and biomolecular binding of Hg species at subcellular level in methylating and nonmethylating sulfate-reducing bacteria. *Environmental Science and Technology* **46**: 11744-11751.

**Ranchou-Peyruse M, Goñi-Urriza M, Guignard M, Goas M, Ranchou-Peyruse A, Guyoneaud R. 2018.** Pseudodesulfovibrio hydrargyri sp. nov., a mercury-methylating bacterium isolated from a brackish sediment. *International Journal of Systematic and Evolutionary Microbiology* **68**: 1461-1466.

**Ranchou-Peyruse M, Monperrus M, Bridou R, Duran R, Amouroux D, Salvado JC, Guyoneaud R. 2009.** Overview of mercury methylation capacities among anaerobic bacteria including representatives of the sulphate-reducers: Implications for environmental studies. *Geomicrobiology Journal* **26**: 1-8.

**Rapp BJ, Wall JD. 1987.** Genetic transfer in Desulfovibrio desusfuricans. *P. Natl. Acad. Sci. USA.* **84**: 9128-9130.

**Skyllberg U, Bloom PR, Qian J, Lin CM, Bleam WF. 2006.** Complexation of mercury(II) in soil organic matter: EXAFS evidence for linear two-coordination with reduced sulfur groups. *Environmental Science and Technology* **40**: 4174-4180.

**Thomas SA, Catty P, Hazemann JL, Michaud-Soret I, Gaillard JF. 2019.** The role of cysteine and sulfide in the interplay between microbial Hg(ii) uptake and sulfur metabolism. *Metallomics* **11**: 1219-1229.

**Weimer PJ, Vankavelaar MJ, Michel CB, Ng TK. 1988.** Effect of phosphate on the corrosion of carbon-steel and on the composition of corrosion products in 2-stage continuous cultures of desulfovibrio desulfuricans. *Applied and Environmental Microbiology* **54**: 386-396.
